# A Review of the Various Roles and Participation Levels of B-Cells in Non-Infectious Uveitis

**DOI:** 10.3389/fimmu.2021.676046

**Published:** 2021-05-14

**Authors:** Lei Zhu, Binyao Chen, Wenru Su

**Affiliations:** State Key Laboratory of Ophthalmology, Zhongshan Ophthalmic Center, Sun Yat-sen University, Guangzhou, China

**Keywords:** uveitis, B cell, autoimmune, B-cell depletion, Rituximab

## Abstract

Non-infectious uveitis is an inflammatory disorder of the eye that accounts for severe visual loss without evident infectious agents. While T cells are supposed to dominate the induction of inflammation in non-infectious uveitis, the role of B cells in the pathogenesis of this disease is obscure. Therefore, this review aimed to discuss diverse B-cell participation in different non-infectious uveitides and their roles in the pathogenesis of this disease as well as the mechanism of action of rituximab. Increasing evidence from experimental models and human non-infectious uveitis has suggested the participation of B cells in non-infectious uveitis. The participation levels vary in different uveitides. Furthermore, B cells play multiple roles in the pathogenic mechanisms. B cells produce autoantibodies, regulate T cell responses *via* antibody-independent functions, and constitute ectopic lymphoid structures. Regulatory B cells perform pivotal anti-inflammatory functions in non-infectious uveitis. Rituximab may work by depleting pro-inflammatory B cells and restoring the quantity and function of regulatory B cells in this disease. Identifying the levels of B-cell participation and the associated roles is beneficial for optimizing therapy. Diversified experimental model choices and emerging tools and/or methods are conducive for future studies on this topic.

## Introduction

Uveitis is an inflammatory disorder of the eye (1). Although it has a relatively low prevalence, it is a major cause of severe visual impairment accounting for 10–20% of visual loss worldwide, and up to 35% of patients with uveitis suffer from effects ranging from severe visual loss to legal blindness (2, 3). It is classified into either infectious or non-infectious uveitis. Non-infectious uveitis is believed to be autoimmune or immune-mediated (4) and is the more prevalent subtype in developed countries (5). Corticosteroids have always been the cornerstone of non-infectious uveitis treatment, albeit with serious side effects due to long-term and high-dose use (6). Immunosuppressants lack universal efficacy and need to be combined with systemic steroids to maintain disease control (7). Biologics are promising therapies that help overcome these handicaps, but a deeper understanding of the pathogenic components involved in non-infectious uveitis is required for improving the precision of targeting.

Non-infectious uveitis is mainly regarded as a T cell-induced disease, and the role of B cells in the pathogenesis of this disease is not yet fully understood (8). Rituximab is a type of B-cell depletion therapy that also includes the humanized B cell-activating factor (BAFF)-targeting monoclonal antibody, Belimumab (9), or the humanized anti-CD22 antibody, Epratuzumab (10). Rituximab treatment has been shown to be useful for treating several human non-infectious uveitides (11, 12) which indicates the participation of B cells in this inflammatory disease; however, this does not conclusively elucidate the role of B cells in the disease. Additionally, the different participation levels of B cells in the disease has not been extensively studied. Therefore, in this review, we explored the different participation levels of B cells and their roles in the pathogenesis of this inflammatory disease. The possible mode of action of rituximab in non-infectious uveitis is also discussed. Identifying the participation of B cells in non-infectious uveitis will help to advance the development or clinical use of medicines targeting B cell or B cell-associated molecules to improve the prognosis of patients.

## Non-Infectious Uveitis

### Definition and Classification

Uveitis is an inflammatory disorder of the eye and involves the vascular uveal tract, retina, optic nerve, and vitreous (1, 13). Etiologically, uveitis is simply classified as infectious uveitis if obvious infectious agents are present, or as non-infectious uveitis if it is suspected to be autoimmune or immune mediated (4). Occasionally, infections may be a potential cause of non-infectious uveitis (14). Non-infectious uveitis is the major focus of this review. Clinically, uveitis is classified anatomically as anterior uveitis (involving the iritis, ciliary body), intermediate uveitis (involving the pars plana, vitreous, and peripheral retina), posterior uveitis (involving the retina and choroid), and panuveitis (involving all ocular tissue) (15). Uveitis can be confined to the eye or be a part of systemic diseases with complex symptoms and varied etiology (16). In approximately 50% of patients with uveitis, a systemic disease will be identified (17) and in the remaining patients, idiopathic uveitis is diagnosed. Common causes of non-infectious uveitis include human leukocyte antigen (HLA)-B27 associated anterior uveitis, Vogt-Koyanagi-Harada syndrome (VKH), sympathetic ophthalmia, Behçet disease, sarcoidosis, Fuchs uveitis syndrome, and multifocal choroiditis (14). Juvenile idiopathic arthritis-associated uveitis (JIAU) is the most common non-infectious uveitis in children in the developed world (18). Therefore, the following content associated with human non-infectious uveitis will be focused on the uveitides discussed above.

### Therapy

Corticosteroids are the mainstay of therapy for non-infectious uveitis. In patients with acute and anterior uveitis, topical corticosteroids and cycloplegic and/or mydriatic agents may help to achieve good results. Unfortunately, patients with chronic, intermediate, posterior uveitis, or panuveitis require more aggressive therapy. Systemic steroids are initially administered, followed by immunosuppressants such as methotrexate and mycophenolate if further anti-inflammatory intervention is required (17, 19). Corticosteroids and conventional immunosuppressants frequently cause side effects in patients (7). Fortunately, biologics may be a remedial choice for patients with refractory non-infectious uveitis. Anti-tumor necrosis factor (TNF) therapy was determined to be useful for controlling several recalcitrant uveitides (20, 21); IL-6 inhibitors also showed efficacy in refractory JIA-associated uveitis, Behcet’s uveitis and so on (22, 23). Significantly, a growing number of reports have supported the use of rituximab, a type of B-cell depletion therapy, for refractory non-infectious uveitis (11, 12); this supports the hypothesis that B cells contribute to the pathogenesis of non-infectious uveitis. Deeper insights into major pro-inflammatory and anti-inflammatory components involved in the pathogenesis of non-infectious uveitis are conducive for optimizing biologic therapy in this disease.

### Experimental Models for Non-Infectious Uveitis

Experimental autoimmune uveitis (EAU) is the most widely studied disease model for human non-infectious uveitis (24). In EAU, immune responses targeting the neural retina and related tissue are mainly induced by T cells (25, 26). Both active immunization of retinal antigens and adoptive transfer of autoreactive T cells can induce EAU in genetically susceptible animals (27). In addition, spontaneous EAU can be established by gene engineering in animals (16). The most well-known retinal autoantigens are interphotoreceptor retinoid-binding protein (IRBP) and soluble antigen (S-Ag) (25). EAU can be established in a variety of animals such as primates, rats, and mice, among others. Of these, mice and rats are the most widely used animals (28). Experimental models recapitulating different uveitides are pivotal for exploring the pathogenic mechanisms of the disease (26). However, no single animal model represents the complete spectrum of human non-infectious uveitis (26). Some discrepancies inevitably exist between EAU and human non-infectious uveitis. For example, T cells tend to target a single retinal antigen in EAU whereas human non-infectious uveitis is likely induced by various T cell populations (29). For the most widely used experimental animal, the development, phenotypes, and immunoglobulins produced by B cells of mice are significantly different from B cells of humans (7). The choroid is much thicker in the eyes of humans than in mice. A thicker choroid might contribute to the development of ectopic lymphoid-like structures (ELSs) which are conducive for antibody production (29). These findings indicate the limitation in EAU to fully represent human non-infectious uveitis. Indeed, no adequate animal model can currently represent the pathogenic mechanisms of JIAU (30).

### Pathogenic Mechanisms Derived From Studies on the EAU Model

**Figure 1:**
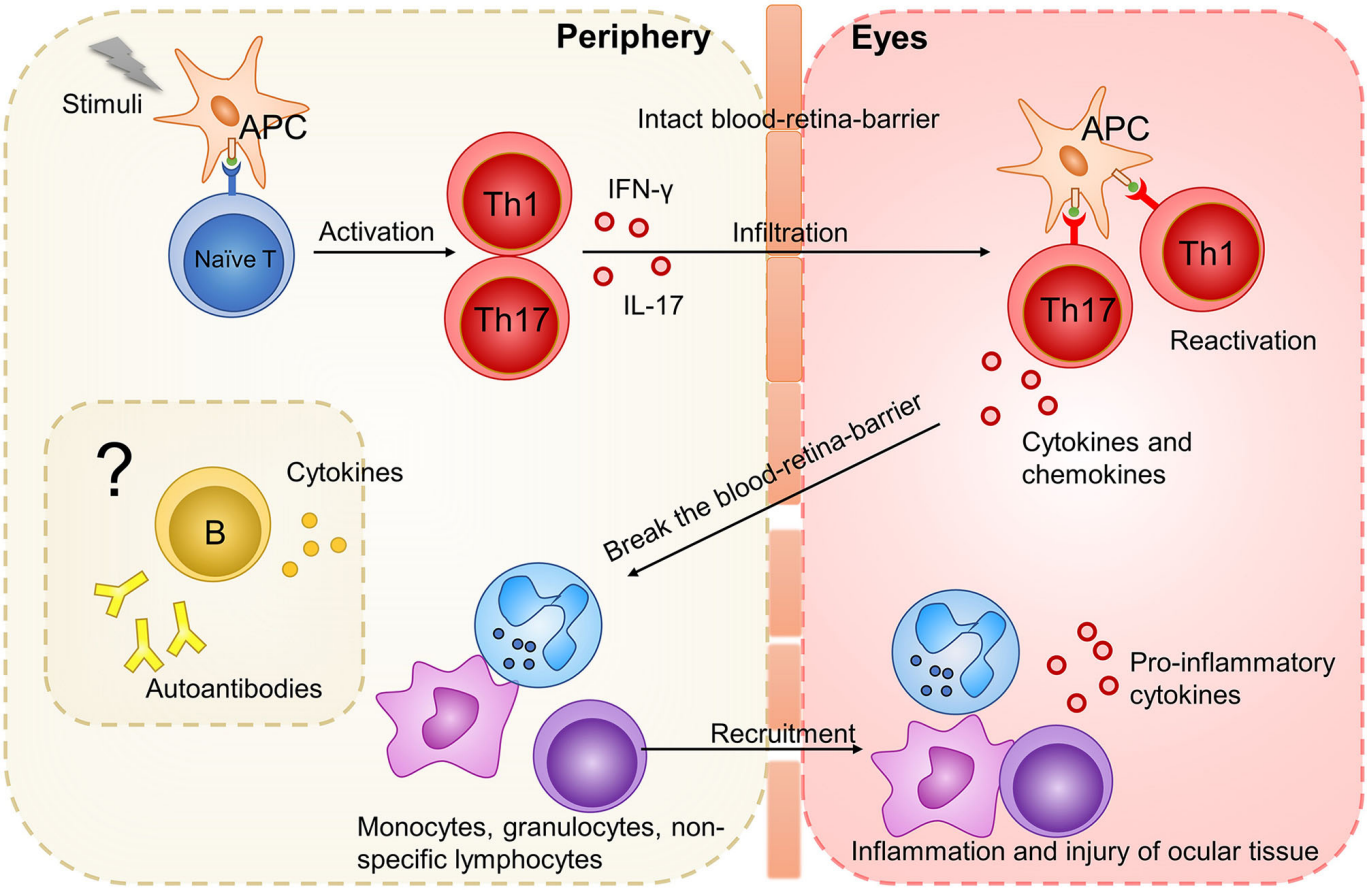
The initiation of uveitis based on studies on EAU model. Stimuli (such as IRBP, pathogens with antigen mimicking ocular antigen, ocular autoantigen released from eyes) activate APC. APC present antigen to naive T cells and promote their differentiation into Th1 and Th17 cells. These activated Th1 and Th17 cells could infiltrate the intact blood-retina-barrier and be reactivated by cross-reactive ocular autoantigen. After reactivation, Th1 and Th17 cells secrete cytokines and chemokines, break the blood-retina-barrier, and attract inflammatory cells such as monocytes, granulocytes and non-specific lymphocytes. These infiltrating inflammatory cells develop inflammation and further destroy the ocular tissue. How B cells function in this process is rarely mentioned.

Studies on EAU demonstrated that non-infectious uveitis is a T helper (Th)1/Th17 cell-driven disease (8). Activated retinal antigen-specific Th1 or Th17 cells can pass the blood-retina-barrier, break it and secrete cytokines and chemokines to attract inflammatory cells including granulocytes, macrophages/monocytes, and non-specific lymphocytes into eyes to develop inflammation and destroy the ocular tissue (16, 28) (**Figure 1**). Both Th1 and Th17 cells can independently transfer the disease to naïve mice, and the type that dominates the disease depends on the model and the method from which the disease is induced (16, 31). Regarding cytokines, interferon (IFN)-γ and interleukin (IL)-17 dominate Th1- and Th17-mediated inflammation, respectively. Increased concentrations of TNF-α in tissue drive T cell responses and macrophage activation in non-infectious uveitis (32). In contrast, IL-10 and transforming growth factor (TGF)-β are pivotal anti-inflammatory cytokines mainly secreted by regulatory T cells (Tregs) in non-infectious uveitis (33). For B cells which play a significant role in autoimmunity, their participation in the pathogenesis of uveitis is rarely mentioned.

However, some studies show results contradicting the pathogenic mechanisms derived from EAU. For example, IFN-γ knockout mice developed even more severe uveitis than did wild-type mice (34). Phase III dose-dependent studies for secukinumab (human antibody targeting against IL-17A) in uveitis failed to show significantly better efficacy than the placebo (35). This discrepancy might be attributed to the differences between EAU and human non-infectious uveitis as we discussed above, and the differences among various experimental models. According to a review published by Caspi et al, many experimental models with varied features have been established (16). Therefore, the current pathogenic mechanisms derived from EAU might be incomplete to apply to all experimental models and human non-infectious uveitis. It is tempting to assume that other factors may be involved in the pathogenesis of non-infectious uveitis in specific experimental models and patients. We can speculate that these factors are B cells because evident B cell infiltration was found in both experimental models and humans with non-infectious uveitis (36–38).

## Participation of B Cells in Non-Infectious Uveitis

In humans, B cells develop from hematopoietic stem cells in the bone marrow. After an intricate developmental process, B cells ultimately develop into plasma cells that secrete antibodies or memory B cells (39). B cells are pivotal components of both humoral immunity and autoimmunity because of their exclusive ability to produce antibodies (40). Other antibody-independent capacities have also received a lot of attention. The pathogenic mechanisms derived from EAU might be incomplete to apply to all experimental models and humans with uveitides as alluded earlier. It is rational to speculate that the involvement of B cells in non-infectious uveitis might be the missing part of the pathogenic mechanisms derived from EAU. Increasing evidence from experimental models and human non-infectious uveitis also supports the involvement of B cells in non-infectious uveitis.

### B-Cell Participation Varied With Different Uveitides

Non-infectious uveitis is a group of heterogeneous diseases with diverse etiologies. Differences exist between EAU and humans non-infectious uveitis, and the pathogenic mechanisms may also be different, as discussed above. Seemingly, differences also exist in multiple clinical subtypes of human non-infectious uveitis. Cytokine and chemokine profiles were found to be different between human non-infectious uveitis subtypes (41, 42). Lymphocyte population (CD4+ and CD8+ T cells and CD19+ B cells) infiltrating the vitreous also varied with uveitis etiologies (43). Therefore, B-cell participation may not only differ between EAU and human non-infectious uveitis, but also varies with distinct human non-infectious uveitides. Accordingly, we provide evidence of B-cell participation in animal models and several common human non-infectious uveitides to explore diverse B-cell participation in EAU and various human non-infectious uveitides (**Table 1**).

**Table 1:** Evidence of B-cell participation in different uveitides.

Different uveitides	Evidence	Reference
**EAU induced in rats or mice**	a. Number of B cells that infiltrated into the eyes was lower than that of T cells. Increased B cell numbers correlated with extended disease duration and severity	(44–46)
	b. Transferring T cells with immune serum exacerbated EAU more than did transferring T cells alone	(25)
**EAU induced in primates**	a. The number of infiltrating B cells and T cells in ocular tissue was similar	(38)
**Juvenile idiopathic arthritis**	a. Plasma cells predominantly constituted the infiltrates with focal aggregates of CD20 positive cells.	(36, 47–50)
	b. Elevated immunoglobulin in the vitreous fluid and anterior chamber exudate	(36, 51)
	c. Upregulated B cell-specific genes and proteins in the iris tissue	(30)
	d. Anti-ocular serum antibodies were more frequently detected	(52)
	e. A patient automatically recovered from uveitis after developing common variable immunodeficiency	(53)
**Sympathetic ophthalmia**	a. In most cases, T cells were dominantly infiltrated into the eyes. B cells outnumbered T cells in four out of 29 eyes and all nine eyes in two independent reports. The predominant B cells correlated with longer disease duration	(37, 54, 55)
**Behçet disease**	a. Small number of B cells, plasma cells, and antibodies in the aqueous humor and ocular tissue	(56, 57)
	b. Modified B-cell function in the serum: increased activated, memory, and spontaneous Ig-secreting B cells; upregulated B cell function indicated by the gene expression of peripheral blood cells; elevated serum immunoglobin levels	(58–61)
**Vogt-Koyanagi-Harada syndrome (VKH)**	a. The number of B cells in the ocular tissue is similar to that in Behçet disease	(62, 63)
	b. Antibodies against ocular structures in the serum	(64)
**Sarcoidosis**	a. The number of B cells in the ocular tissue is similar to that in Behçet disease	(65)
**Idiopathic uveitis**	a. The number of B cells in ocular tissue is similar to that in Behçet disease	(66)
**Multifocal choroiditis**	a. Infiltrating B cells outnumbered T cells in inflammatory sites	(67, 68)
**Fuchs’ heterochromic cyclitis**	a. Oligoclonal IgG bands were found in the aqueous humor but not in the serum	(69)
**a. The number of B cells in the aqueous humor collected from patients with idiopathic uveitis is less than that from patients with systemic diseases**		(70)
**b. Levels of B cell-associated molecules (BAFF, APRIL, CXCL13) were significantly higher in the aqueous humor of patients with active uveitis than in healthy controls. APRIL and CXCL13 levels were higher in patients with granulomatous uveitis than in patients with non-granulomatous uveitis.**		(41, 42, 71)

a–e: the sequence of evidence in each uveitis.

### B-Cell Participation in EAU

The number of B cells infiltrating the ocular tissue during uveitis is different depending on the experimental model used. The cell population in the eye also changes over time: while neutrophils dominate the acute stage of retinal inflammation, lymphocytes dominate the chronic stage (72). In most uveitis-induced rats or mice, a small number of B cells and plasma cells were reported to infiltrate in inflammation (a report showed a concrete ratio of 1:4 between CD19+ B and CD4+ T cells), and an increase in the number of B cells is associated with extended disease duration and aggravated severity (44–46). These data suggest that B cells participate in the pathogenesis of EAU, but only play a minor role in most cases. Chronic inflammation may lead to B cell infiltration. The increased B cell number may lead to persistent disease owing to antibodies produced by plasma cells or other pro-inflammatory functions of B cells. However, in another primate model, a ratio of 1.4:1 between infiltrating CD3+ T and CD19/CD22+ B cells has been found during inflammation (38). The proportion of infiltrating B cells is much higher than that in rats or mice, suggesting that B cells may participate in human non-infectious uveitis more actively than we previously considered, because primates are more semblable to humans. Regarding autoantibodies, which are exclusively secreted by B cells, although transfer of immune serum alone fails to transfer uveitis to recipients, transfer of T cells with immune serum is associated with a higher disease severity compared to the transfer of T cells alone. This suggests that circulating antibodies may be too large to penetrate the blood-eye barrier (73) and they can gain access and exacerbate EAU after this barrier is disrupted by T cells (25). It also verified the pro-inflammatory function of autoantibodies against ocular tissue during non-infectious uveitis.

### B-Cell Involvement in Several Common Human Uveitides

In juvenile idiopathic arthritis (JIA), uveitis is the most common extra-articular manifestation. The morbidity of uveitis in children with JIA is approximately 10%, among which non-granulomatous anterior uveitis is the most common form (74, 75). Unlike that observed in EAU, a primary B cell infiltrating process was observed in the ocular tissue of patients with juvenile idiopathic arthritis-associated uveitis (JIAU) using immunohistochemical studies. Plasma cells predominantly constitute the inflammation in the iris and ciliary body with a few T cells and macrophages (47–50). Within these plasma cells, one study showed IgM+ plasma cells were the dominant while the other showed the infiltrate is primarily made up of IgG+ plasma cells (48, 50). Focal aggregates of CD20-positive cells have also been shown (36). Correspondingly, elevated amounts of immunoglobulin (Ig) were observed in the vitreous fluid (51) as well as in the anterior chamber exudate, suggesting local production of antibodies (36). Intraocularly upregulated B cell-specific genes and proteins, especially several Ig components in iris tissue, ulteriorly evidence that B cells participate in the pathogenesis of JIAU (30). Regarding serum antibodies, using indirect immunohistochemistry, anti-ocular serum antibodies can be detected more frequently in JIAU groups than in control groups and predominantly bind to the iris and ciliary body, consistent with the major inflammatory sites (52). Finally, a previous case report showed that a patient automatically recovered from JIAU after developing common variable immunodeficiency, in which defective antibody formation is the most common feature with B cell differentiation failure (53). Overall, JIAU appears to be a B cell (especially plasma cell)-mediated disease. Antibodies produced both in the eye and in the bloodstream exert pathogenic effects.

Sympathetic ophthalmia (SO) is defined as bilateral granulomatous panuveitis following penetrating ocular injury to one eye (76). The results of immunohistochemical studies of choroidal infiltrates of the SO are diverse. In most cases, the choroid is infiltrated mainly by T cells with a small number of B cells and plasma cells (54). However, a study that was conducted using 29 cases showed the predominance of CD45RO+ T cells in choroidal infiltrate in 20 cases and that of CD20+ B cells in four cases; it showed equal numbers of T cells and B cells in the remaining five cases (37). In another study, CD20+ B cells and CD68+ macrophages were more abundant than CD3+ T cells in all nine cases (55). The predominance of B cells correlated with a longer duration of the disease (37); similar results were observed in animal model-based studies. It raises the question of whether an increase in the number of infiltrating B cells increases chronic disease duration or whether chronic inflammatory lesions of the eye attract more B cells. Alternatively, it is a mutually reinforcing vicious circle that can be interrupted by B-cell depletion therapy.

Behçet disease (BD) is characterized by occlusive vasculitis and can affect many different systems. Ocular manifestations are usually bilateral non-granulomatous panuveitis and occlusive retinal vasculitis (54). Several studies reported uveitis in 43-65% of patients with BD (58). In most cases, B cells, plasma cells, and antibodies can be found in the aqueous humor and ocular tissue in patients with BD, although T cells always outnumbered B cells (56, 57). In serum, a modified B cell function has been reported in patients with active BD. Although the total CD19+ B number was unchanged, activated and memory B cell subsets as well as spontaneous immunoglobulin(Ig)-secreting B cells increased (59). However, another study found B cells in serum was less in patients than in controls, mostly due to decreased CD27+ memory B cells expressing IgM, IgG and IgA (77). The relocation of memory B cells into the site of inflammation may account for the deviation found in the blood, since the demonstrated abundance of B cells in the inflammation (77). Serum immunoglobulin levels are also elevated (58). Gene expression profiling of peripheral blood cells also indicated that B cells activated and produced autoantibodies during BD (60, 61). However, whether elevated circulating antibodies and abnormal B cells in serum are associated with ocular manifestation are indeterminate. Comparative tests between patients with BD with or without uveitis may help to answer this question.

In other clinical entities of uveitis, evidence of B-cell participation is rare and derived from earlier research. As observed in eyes with BD, immunohistochemical studies showed the predominance of T cells and the presence of a relatively fewer number of B cells and plasma cells in ocular infiltrates of patients with VKH (62, 63), sarcoidosis (65), and idiopathic uveitis (66). In patients with VKH, serum autoantibodies against gangliosides, outer segments of the photoreceptors, and Müller cells have been detected (64). Limited data are available on multifocal choroiditis, which show that infiltrating B cells outnumbered T cells in inflammatory sites (67, 68). In patients with Fuchs’ heterochromic cyclitis, oligoclonal IgG bands were found in the aqueous humor but not in the serum, suggesting the intraocular production of IgG with restricted specificity (69). However, the significance of the IgG bands is still unknown.

### Comparison of B-Cell Participation in Diverse Non-Infectious Uveitides

We have provided evidence of B-cell participation in EAU and several common human non-infectious uveitides in terms of the number of B cells and plasma cells infiltrating the ocular tissue; antibodies found in eyes; gene and protein expression profiles of B cells; and serum components including changes in the numbers of peripheral B cells and antibodies. From these data, diverse B-cell participation in different cases of non-infectious uveitis can be concluded. In most cases, B cells play a minor role in EAU, but exceptions still exist. In human non-infectious uveitis, B cells seem to highly contribute to the pathogenic mechanisms of JIAU, SO, and multifocal choroiditis. Particularly in JIAU, strong evidence confirmed the pro-inflammatory functions of B cells in uveitis by producing antibodies. In other human non-infectious uveitides, B cells appear to play a minor role in the pathogenesis. However, as the number of cases in each experiment is relatively small and the accessible ocular tissue is usually derived from advanced diseases, it is difficult to draw a precise conclusion, especially regarding the onset of the disease.

Several comparative tests have also explored this subject. A report revealed that the number of CD19+ B cells in the aqueous humor derived from patients with idiopathic uveitis (including anterior uveitis and panuveitis) is lower than that in patients with systemic disease-associated uveitis (including ankylosing spondylitis, sarcoidosis, Behçet’s disease) (70). Two experiments compared the involvement of B cells in granulomas and non-granulomas uveitis by analyzing B cell-associated molecules. BAFF and a proliferation-inducing ligand (APRIL) are survival factors of B cells and plasma cells (78, 79). B cell chemoattractant CXCL13 can attract B cells and follicular helper T cells to form ectopic lymphoid structures (ELSs) (80, 81). Levels of APRIL, BAFF, and CXCL13 were significantly higher in aqueous humor samples collected from patients with active uveitis than in healthy controls (CXCL13 was not detected) (41, 71) suggesting that B cells are involved in the pathogenesis of uveitis. Additionally, APRIL and CXCL13 levels were significantly higher in patients with granulomatous uveitis (sarcoidosis and VKH) than in patients with non-granulomatous uveitis (BD and HLA-B27 associated anterior uveitis) (41, 42, 71). These higher levels of molecules suggest that B cells may be pathogenetically more important in granulomatous uveitis (42). This hypothesis has a limited application scope. The participation of B cells in SO, usually granulomatous uveitis, confirmed it, whereas it was inconsistent with the high involvement of B cells in JIAU, usually non-granulomatous uveitis. Notably, even in the same uveitis subtype, the participation of B cells may change with the disease duration, as discussed in EAU and SO. Predominance of B cells tends to correlate with a long disease duration.

Overall, B-cell participation in non-infectious uveitis differs on a case-to-case basis. Identifying the types of uveitides that show high B cell participation may be beneficial for selecting patients suffering from refractory non-infectious uveitis for B-cell depletion therapy or other biologics targeting B-cell-related molecules. According to the concrete mechanisms of B-cell participation, the elevated antibodies found in the ocular tissues or sera of patients with non-infectious uveitis demonstrate that B cells promote inflammation in patients by producing antibodies. However, other mechanisms of B cell-participation in non-infectious uveitis are not shown by the above evidence. This will be discussed in detail in the subsequent section.

## B Cells Play Multiple Roles in Autoimmunity and Non-Infectious Uveitis

B cells have been viewed as central contributors to autoimmunity because of their ability to produce antibodies (40). The antibody-independent functions of B cells have received attention after clinical trials supported the efficacy of rituximab in several autoimmune diseases, including those considered to primarily involve T cells (82, 83). Rituximab is an anti-CD20 monoclonal antibody (84) and has no major effect on immunoglobulin production because plasma cells do not express CD20 (85–87). B cells can also help develop ectopic lymphoid-like structures conducive to antibody production. Moreover, a group of regulatory B cells (Bregs), which can inhibit inflammation, have recently captured researchers’ attention (88). Many studies have identified multiple functions of B cells in various autoimmune diseases. In non-infectious uveitis, these functions of B cells were also evident. Hence, we will discuss the multiple functions of B cells in autoimmunity and highlight their roles in non-infectious uveitis in this part.

### B Cells Promote Inflammation by Producing Autoantibodies

Autoantibodies produced by plasma cells promote inflammation in multiple ways, as reviewed in other studies (89, 90): autoantibodies lead to direct cell lysis; antigen-antibody immune complexes that are deposited in organs or tissues, activate complements and Fc receptor-bearing leukocytes including granulocytes and macrophages to extend inflammation; binding of autoantibodies to receptors on cell surfaces could block or excessively upregulate their functions. These mechanisms have been found in many autoimmune diseases such as systemic lupus erythematosus (SLE), autoimmune hemolytic anemia, and Grave’s disease.

In non-infectious uveitis, the existence of autoantibodies against ocular tissues has been confirmed in several different uveitides. Especially in JIAU, autoantibodies were found both in the eye and in the bloodstream. Antibodies cannot pass through the complete blood-eye barrier (73). Thus, antibodies in the circulation may impair ocular tissue only after the diseases are established and the blood-eye barrier is broken. In addition, antibodies produced locally may function earlier. The pro-inflammatory function of antibodies was verified by the study that transferred T cells with immune serum as alluded earlier. However, the above study did not demonstrate the mechanism of inflammation during non-infectious uveitis. We hypothesized that antibodies that promote inflammation, as evidenced in other autoimmune diseases, also exist in non-infectious uveitis. More research is needed to explore the concrete mechanisms of how antibodies function during non-infectious uveitis.

### B Cells Regulate T Cell Response *via* Their Autoantibody-Independent Function

B cells can regulate the proliferation and differentiation of T cells *via* antigen presentation, co-stimulation, and cytokine secretion. B cells present antigens to T cells *via* major histocompatibility complex (MHC)-I or II (91) and express costimulatory molecules, including CD80, CD86, and CD40 (92, 93). These two functions have been proven using genetically engineered mice with autoimmune diseases, in which only B cells lack MHC-II molecules or costimulatory molecules. In these mice, autoantigen-induced disease establishment was abrogated, T cell clonal expansion was significantly reduced, and the differentiation of T cells into cytokine-secreting effector T cells and memory T cells was impaired (94, 95). T cells from these mice lost the capacity to transfer autoimmune disease (93). As cytokine-producers, B cells produce proinflammatory cytokines such as TNF-α, IFN-γ, IL-6, and IL-12 (96). B cells have been identified as major producers of IL-6 in disease models for systemic lupus erythematosus (SLE) and multiple sclerosis (MS) (97, 98). *In vitro* experiments also implied that B cells can produce GM-CSF to activate myeloid cells (99). These cytokines can influence the activities of various cells, including T cells (100). B cells modulating T cell response *via* antibody-independent pathways is also indirectly proven by decreased tissue CD4 T cell numbers (101), Th1/Th17 responses, and increased regulatory T cell (Treg) numbers (102) after rituximab treatment in several autoimmune diseases.

With regard to non-infectious uveitis, rituximab has been shown to be effective in patients with chronic anterior uveitis (103), refractory Behçet disease-associated uveitis (104), JIA (105, 106), VKH (11, 107, 108), multifocal choroiditis (109), and other more rare uveitides (110). The patients involved in these case reports or case series mainly developed severe uveitis with prolonged disease duration and resistance to topical and systemic corticosteroids, immunosuppressors, TNF-α inhibitors, or other biologics. Resistance to some of the classical drugs and the response to rituximab indirectly suggest that B cells participate in the pathogenesis of non-infectious uveitis in autoantibody-independent ways, particularly in the late phase of diseases. However, it is difficult to discuss how B cells participate in the onset of the disease since rituximab is not administered to patients with new-onset non-infectious uveitis. Additionally, the efficacy studies of rituximab only demonstrate its overall autoantibody-independent function; specific functions involving antigen presentation, co-stimulation, and cytokine secretion have still not been extensively studied. Corresponding studies on preclinical models for non-infectious uveitis are lacking. We can refer to studies conducted on other autoimmune diseases that induce EAU in mice in which only B cells lack MHC-II molecules or costimulatory molecules. In this way, we may obtain more direct evidence and explore the autoantibody-independent function of B cells at the onset of the disease.

### B Cells Constitute ELSs

ELSs, also called tertiary lymphoid organs, are structurally and functionally similar to secondary lymphoid organs (111, 112). ELSs can develop within chronic inflamed organs such as the joints of patients with rheumatoid arthritis (RA) (113) and the central nervous system of patients with MS (114). ELSs mainly consists of B cells (112). It contains anatomically distinct but adjacent B and T cell areas and germinal centers where T follicular helper cells (Tfh) and follicular dendritic cells (FDC) help B cells develop into long-living plasma cells and generate antibodies (111, 115). In most cases, ELSs are associated with a severe disease and local production of autoantibodies (116–118).

ELSs were first reported in three out of 11 patients with chronic non-infectious uveitis 4 years ago (66). In addition to B cells, follicular B cells, macrophages, and plasma cells are components of ELSs (66). ELSs were also found in experimental models (45, 119, 120). In IRBP TCR transgenic R161H mice, approximately 40% of mice developed ELSs. More detailed cell composition was characterized in this experimental model; ELSs were found to comprise approximately 30% CD4+ T cells, 10% CD8+ T cells, and 60% B220+ B cells. CD138+/B220+ plasma cells were found in large well-developed ELSs, which usually occurs in the late stage of the disease (120). The function of ELSs in ocular tissue is poorly understood. In the same experimental model, when ELSs spread out, increased loss of visual function occurred in mice. In addition, mice with ELSs had higher levels of serum antibodies against IRBP than did those without ELSs. These may result from the local generation of autoantibodies by plasma cells in the well-developed ELSs (120). Overall, B cells constitute ELSs, which may contribute to an increase in the severity of the disease and local production of autoantibodies in non-infectious uveitis.

### B cells Inhibit Autoimmunity Prominently by Secreting Anti-Inflammatory Cytokines

In the past two decades, regulatory B cells (Bregs) have been identified as suppressive components of autoimmunity (40). Bregs are relatively rare and account for less than 10% of B cells in circulation in healthy people (121). Although there is no unique surface marker that exclusively identifies Bregs, most of them express CD24, CD1d, CD38, and CD5 (122–124). They possess the same feature of suppressing immune responses dominantly *via* secretion of IL-10; thus, the secretion of IL-10 serves as a marker of Bregs (40). In addition to IL-10, IL-35 has been recognized as another key immunoregulatory cytokine produced by Bregs (88). IL-10 and IL-35 suppress inflammation by several mechanisms, including inhibition of Th1, Th17, and monocytes secreting INF-γ, IL-17, and TNF-α and promoting the generation of Tregs (125, 126). In particular, IL-35 can induce Bregs generation and ultimately promote IL-10 and IL-35 production (126). Furthermore, B cells could regulate autoimmunity *via* upregulating the inhibitory receptor Programmed Cell Death-Ligand 1 (PD-L1) (127) or killing CD4+ T cells by FasL/Fas-dependent mechanisms (128).

**Figure 2:**
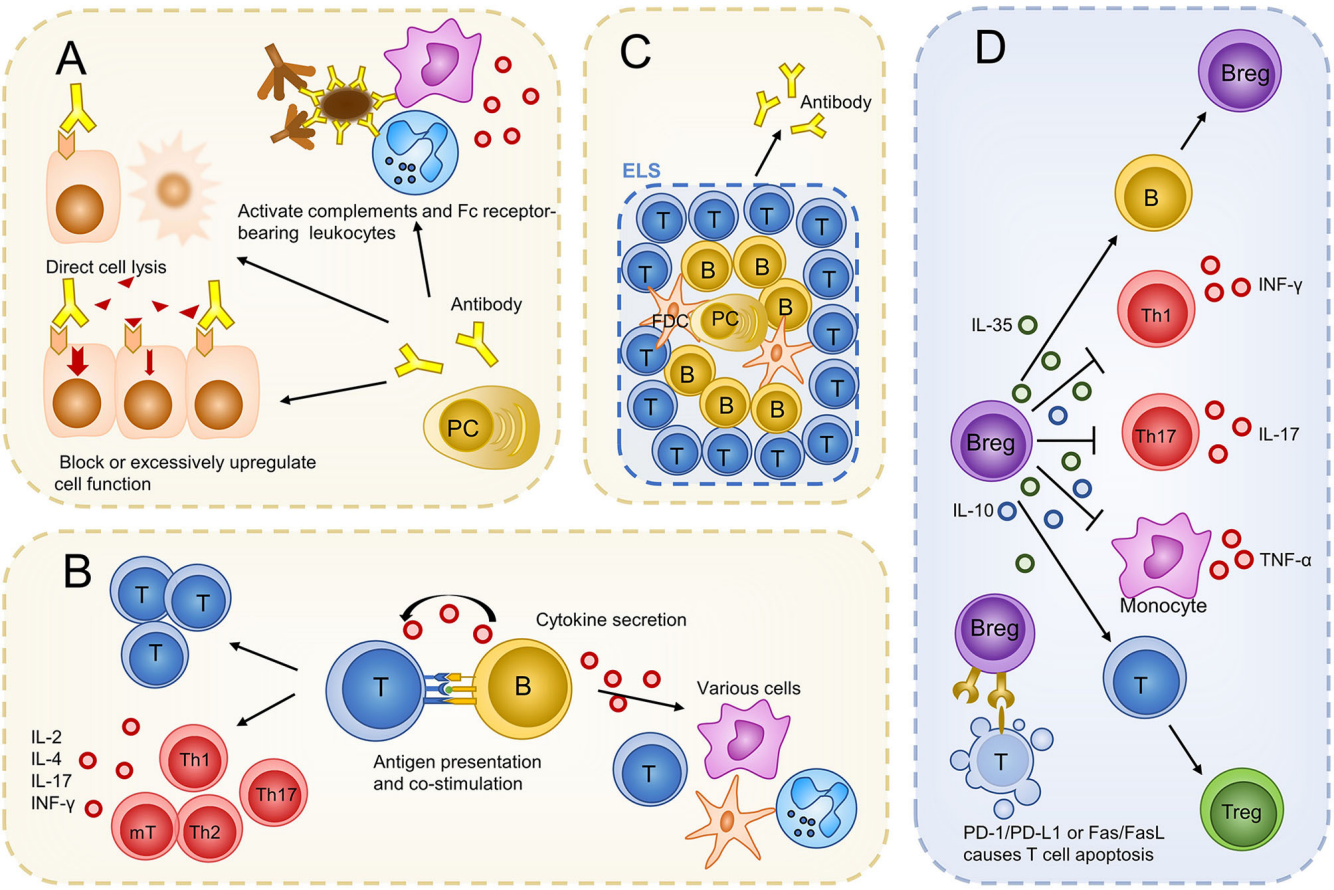
Possible roles of B cells in non-infectious uveitis. B cells evolve into plasma cells and produce antibodies to promote inflammation and damage ocular tissues **(A)**. Via antibody independent functions, B cells regulate the proliferation and differentiation of T cells. Cytokines produced by B cells can also influence other immune cells **(B)**. B cells constitute ELS to produce more autoantibodies **(C)**. Bregs regulate immunity mainly by producing IL-10 and IL-35. Bregs can also kill T cells *via* a PD-1/PD-L1 or Fas/FasL-dependent mechanism **(D)**. PC, plasma cell; T, T cell; B, B cell; mT, memory T cell; Th, T helper cell; FDC, follicular dendritic cell; Tfh, follicular helper T cell; ELS, ectopic lymphoid structures; IL, interleukin; PD-L1, programmed cell death-ligand 1.

In non-infectious uveitis, using flow cytometry analysis, Bregs were found in the retina during EAU and surprisingly accounted for >40% of all B cells detected (46). This proportion was much higher than that in peripheral blood, which was reported to be 10%, and was close to half of all B cells, suggesting that Bregs are positively recruited into the ocular inflammation and that the regulatory function is an important component of B-cell participation in non-infectious uveitis. The functions of IL-10 and IL-35 were also confirmed in non-infectious uveitis. Neutralization of IL-10 with antibody exacerbated EAU and overexpression of IL-10 in the eye ameliorated uveitis (129). Recombinant IL-35 (rIL-35)-treated mice showed mild EAU with an increase in the number of Bregs and IL-35 producing Bregs (i35-Bregs), indicating that IL-35 could suppress uveitis by inducing the expansion of Bregs and i35-Bregs (130). Human recombinant IL-35 inhibited IL-17 and IFN-γ production and induced IL-10 production in peripheral blood mononuclear cells (PBMCs) when co-cultured with PBMCs from patients with VKH (131). Moreover, Bregs, but not Tregs or myeloid cells producing major IL-10 and IL-35 during EAU, have been demonstrated using flow cytometry and intracellular cytokine staining assays (46). It is tempting to speculate that Bregs, but not Tregs, are the major regulatory component during non-infectious uveitis. Overall, B cells play multiple roles in autoimmunity, and these functions have also been verified in non-infectious uveitis (**Figure 2**).

## How Rituximab Works in Non-Infectious Uveitis

**Figure 3:**
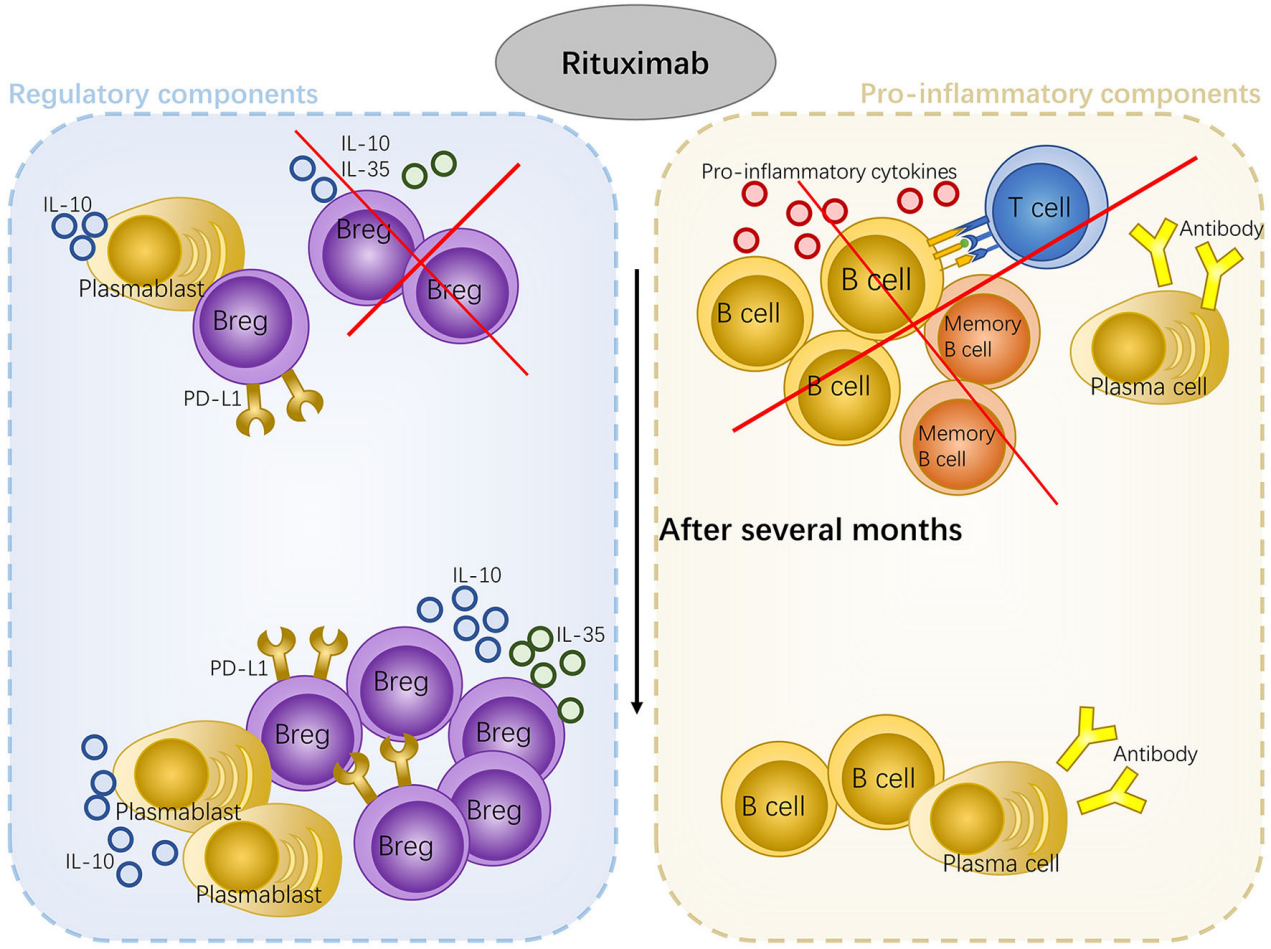
The mechanism of rituximab treatment in uveitis. Rituximab eliminates effector B cells and malfunctioning Bregs as well as induces the restoration of the quantity and function of Bregs. Some regulatory B cell lines resistant to rituximab also expand and contribute to the efficacy of this drug.

B-cell participation in non-infectious uveitis can be separated into two parts: anti-inflammatory and pro-inflammatory components. Rituximab has no major effect on immunoglobulin production. It may work by eliminating the antibody-independent function of B cells, including promoting the T cell response *via* antigen presentation, co-stimulation, and cytokine secretion. Rituximab can also abrogate the establishment of ELSs. However, at the same time, Bregs, the pivotal regulatory components in uveitis, are also eliminated. There is a possibility interpretation for this. The quantity and function of Bregs are adversely affected during non-infectious uveitis. The number of Bregs decreased in the peripheral blood of patients with SLE (123), RA (132), and some other autoimmune diseases (133, 134). In patients with SLE, Bregs produced low levels of IL-10 in response to CD40 and were unable to inflict the Th1 reaction (123). Injured Bregs function was also found in patients with MS (135). Reduced serum levels of IL-35 in patients with active VKH (131)and patients with BD partly confirmed this scenario (136). Repopulation of B cells after B-cell depletion therapy has been reported in patients with neuromyelitis optica (135), RA (137), and pemphigus (138). In neuromyelitis, after rituximab treatment, most circulating B cells were removed. After 6 months, B cells reemerged, and the majority showed the naïve CD27 negative phenotype [CD27 is present on the surface of memory B cells and antibody-secreting B cells (139)]. Newly developed B cells secreted increased levels of IL-10 and decreased levels of lymphotoxin, suggesting that B cells changed toward a more naïve phenotype with a restored Bregs quantity as well as function (135). Therefore, rituximab may also work by restoring the quantity and function of Bregs in non-infectious uveitis. There are other regulatory B cell populations with high resistance to anti-CD20 antibodies. IL-10-producing plasmablasts (140) lack CD20. Bregs expressing high levels of PD-L1 also resist anti-20 therapy primarily due to their high levels of BAFF receptor (BAFF-R) expression (141). The efficacy of rituximab against uveitis may partly come from the expansion of these resistant regulatory B cells. Overall, in addition to eliminating effector B cells that can promote T cell response, rituximab may also restore Bregs quantity and function during non-infectious uveitis (**Figure 3**).

## Discussion

The participation of B cells in non-infectious uveitis has been ascertained by increasing evidence from animal models and several human non-infectious uveitides. Significantly, B cells may participate to a great extent in the pathogenesis of some uveitis subtypes, such as JIAU, and their participation may be limited in the others, as in EAU induced in most experimental models. As in the case of autoimmunity, B cells play multiple roles in the pathogenic mechanisms of non-infectious uveitis. Rituximab is useful for treating several refractory uveitis cases. Its mechanism of action may involve the depletion of pro-inflammatory B cells and restoration of Bregs quantity and function at the same time.

However, there are still some unsolved questions concerning B-cell participation in non-infectious uveitis in need of future exploration. For instance, the following are unclear: the time of participation of B cells in the disease, particularly, if B cells can trigger non-infectious uveitis or if they only function after inflammation has been induced by T cells; the relationship of B cells with disease duration and severity; and the ratio of the anti-inflammatory function to the pro-inflammatory function of B cells during non-infectious uveitis. Clinical studies using ocular tissue or serum samples of patients are not sufficient to discuss these questions. EAU has limitation in fully representing all different human non-infectious uveitides. Thus, diversified experimental model choices could be considered in future exploration. Moreover, although histology, immunohistochemistry, and flow cytometry studies are good tools to identify immune cells in uveitis, it is difficult to use them for testing the dynamic, *in vivo* changes and for obtaining direct evidence of cell function. Consequently, new technologies are supposed to overcome these limitations and thus help to further ascertain B-cell engagement in non-infectious uveitis. For example, *in vivo* immune-cell-specific bioluminescence successfully measures the dynamic intraocular immune cell population during EAU according to the change in luminous intensity (142). Single-cell RNA sequencing is a comprehensive and unbiased approach to investigate cell types and provide information about the function of lymphocytes by analyzing gene expression patterns during non-infectious uveitis (45).

Finally, identifying the participation levels of B cells and their function is conducive for guiding B-cell depletion therapy and for using more targeting biologics in non-infectious uveitis as well as in other autoimmune diseases. Aside from depleting B cells directly, therapies that enhance the function of Bregs as well as drugs that target B cell associated molecules such as BAFF, APRIL and CXCL13 (108) should also be considered in future studies.

## Author Contributions

Conception and design: WS. Drafting and revising of the article: WS, LZ, and BC. Final approval: WS. All authors contributed to the article and approved the submitted version.

## Funding

This work was supported by the National Key Research and Development Program of China (2017YFA0105804).

## Conflict of Interest

The authors declare that the research was conducted in the absence of any commercial or financial relationships that could be construed as a potential conflict of interest.
